# Reliability of pelvic floor muscle strength assessment in healthy continent women

**DOI:** 10.1186/s12894-015-0017-6

**Published:** 2015-04-10

**Authors:** Dulcegleika VB Sartori, Monica O Gameiro, Hamilto A Yamamoto, Paulo R Kawano, Rodrigo Guerra, Carlos R Padovani, João L Amaro

**Affiliations:** Department of Urology, Medical School of Botucatu, São Paulo State University, Botucatu, Brazil; Coordinator of Pelvic Floor Rehabilitation Service, Medical School of Botucat, São Paulo State University, Botucatu, Brazil; Department of Biostatistics, Medical School of Botucatu, São Paulo State University, Botucatu, Brazil; Department of Urology, School of Medicine, São Paulo State University (UNESP), Campus de Rubião Júnior, s/n, 18618-970 Botucatu, SP Brazil

**Keywords:** Gynecological examination, Parity, Pelvic floor, Urinary Incontinence, Reproducibility of results

## Abstract

**Background:**

The aim of this study was to compare pelvic floor muscle (PFM) strength using transvaginal digital palpation in healthy continent women in different age groups, and to compare the inter- and intra-rater reliability of examiners performing anterior and posterior vaginal assessments.

**Methods:**

We prospectively studied 150 healthy multiparous women. They were distributed into four different groups, according to age range: G1 (n = 37), 30–40 years-old; G2 (n = 39), 41–50 years-old; G3 (n = 39), 51–60 years-old; and G4 (n = 35), older than 60 years-old. PFM strength was evaluated using transvaginal digital palpation in the anterior and posterior areas, by 3 different examiners, and graded using a 5-point Amaro’s scale.

**Results:**

There was no statistical difference among the different age ranges, for each grade of PFM strength. There was good intra-rater concordance between anterior and posterior PFM assessment, being 64.7%, 63.3%, and 66.7% for examiners A, B, and C, respectively. The intra-rater concordance level was good for each examiner. However, the inter-rater reliability for two examiners varied from moderate to good.

**Conclusions:**

Age has no effect on PFM strength profiles, in multiparous continent women. There is good concordance between anterior and posterior vaginal PFM strength assessments, but only moderate to good inter-rater reliability of the measurements between two examiners.

## Background

Urinary incontinence (UI) in women is common and prevalence increases with age [1,2]. Damage to the pelvic floor muscle (PFM) can decrease the muscle strength and consequently could result in urinary and fecal incontinence [2]. It has been demonstrated that the weakness of the PFM is significantly higher in incontinent women [3,4] and also that this weakness is worse in women with urge urinary incontinence [5]. According to the International Classifications of Impairments, Disabilities and Handicaps (ICIDH), a nonfunctioning PFM occurs when there is a reduction in force generation and incorrect timing or coordination of muscle contraction [6].

The PFM function can be evaluated using vaginal palpation, visual observation, electromyography, ultrasound, and magnetic resonance imaging [6]. The vaginal palpation is currently used by most physical therapists to assess PFM contraction. However, there has been no systematic research to determine the best method of vaginal palpation to evaluate the pelvic floor contraction [6], and different score systems have been described.

The Brink score [7] and the Laycock PERFECT assessment scheme [8] are commonly used to evaluate PFM function [9]. Some authors have reported that the best reliability is obtained by a digital examination (Brink Score) followed by perineometer evaluation and then by vaginal cone tests in incontinent elderly women [10]. Despite this, other authors have shown poor inter-rater reliability using a modified Oxford scale to assess PFM function [11,12]. A simplified non-validated scale for PFM assessment was proposed by Amaro [13]. On the other hand, some authors have observed that PFM contractions at 50% intensity, in asymptomatic subjects, actually had a gradient of pressure, which increases in the anterior and posterior directions of the vagina, and which is greater than in incontinent patients [4]. This indicates that there is an antero-posterior vaginal pressure profile (VPP) along the vagina, and therefore highlights the importance of assessing PFM strength both at the anterior and posterior regions of the vagina, instead of evaluating it at any random position [4].

It would be interesting to determine the baseline and distribution of force along the vagina of healthy continent women. Despite the number of different studies on the reliability of PFM evaluation, there is no consensus about the most valid and reliable method. Additionally, knowledge about normal PFM evolution with aging is limited.

The aim of this study was to evaluate PFM strength using transvaginal digital palpation (TDP) in healthy multiparous continent women, in different age groups, and to compare anterior and posterior vaginal assessment, establishing examiners’ inter and intra-rater reliability.

## Methods

We prospectively studied 150 healthy multiparous women with an average age of 50 years. All patients were informed about the procedures and study objectives and provided written consent, as approved by the Ethical Committee in Research of Universidade do Sagrado Coração - USC (protocol number: 61/07). Exclusion criteria were UI and/or lower urinary tract symptoms, neurological diseases, previous pelvic surgeries, diabetes mellitus, smoking, and cognitive problems.

The participants were distributed into four different groups according to age range: G1 (n = 37), 30–40 years-old; G2 (n = 39), 41–50 years-old; G3 (n = 39), 51–60 years-old; and G4 (n = 35), older than 60 years-old. Demographic data, such as age, number of deliveries, body mass index (BMI), and physical and sexual activity, were all obtained using a clinical questionnaire. BMI was calculated and classified according to World Health Organization [14] guidelines.

**Figure 1:**
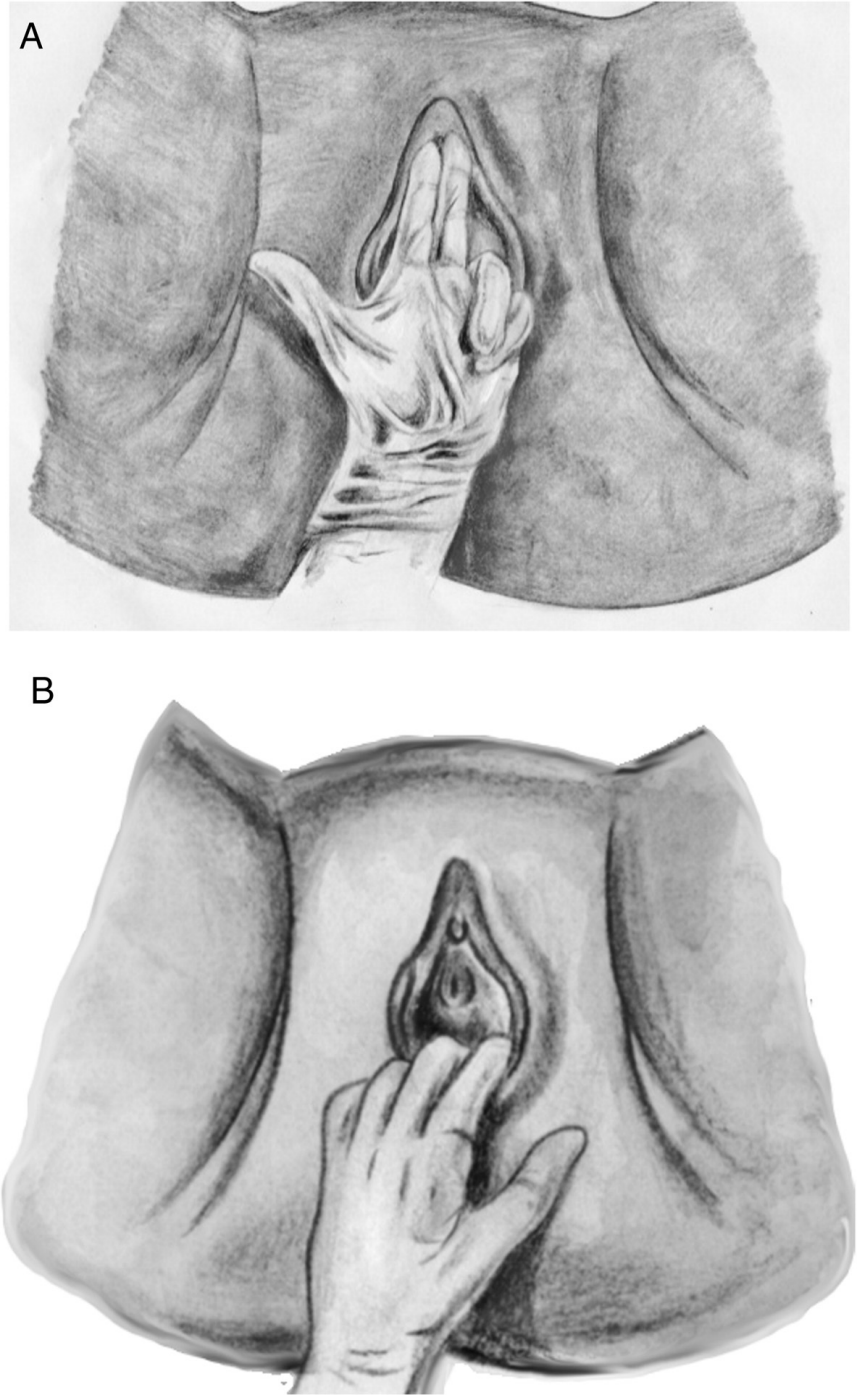
**Bidigital vaginal palpation of the vaginal introitus: Anterior (A) and Posterior (B).**

PFM strength assessments were performed using TDP. The subjects lay in a supine position with a pillow under their heads, with their knees straight and legs abducted. The examiners used their second and third fingers for examination, extended and fully inserted into the vagina, but avoiding any excessive discomfort. The participants were then instructed to contract the pelvic floor muscles against the examiner’s fingers and hold this contraction as long as possible. Contractions at either anterior and posterior regions of the vagina were assessed sequentially, with the same method (Figure 1A and B). Muscle strength was graded using the 4-point Amaro´s Scale: 0 = no contraction, 1 = mild muscular contraction, sustained for less than 3 seconds (s), 2 = moderate muscular contraction, sustained for less than 5 s, and 3 = Normal muscular contraction, sustained for more than 5 s. This classification was tested but not validated [13]. Three experienced physical therapists (more than 1 year since graduation) conducted this study (A, B, and C). They sequentially graded each participant’s PFM strength, both at anterior and posterior vaginal regions, separately from each other. The palpation test was performed in random order of examiner, and the results of each evaluation were kept in sealed envelopes, blinded to the other examiners, in order to avoid influencing their evaluations.

### Statistical analysis

Sample size was calculated for a significance level of 10% and test power of 95%. The characteristics of our health service were also taken into account. We invited three of each four women seen consecutively to enroll. According to these results and considering the range between percentages of answers as the casual error, the minimum of 150 women was established, proportionally distributed in four different age groups.

Data were analyzed using SPSS® software (IBM Corp., Armonk, New York, USA). When the data followed a Gaussian or normal distribution, analysis of variance was used. When the data were not normally distributed, the nonparametric Spearman coefficient and Kruskal–Wallis test were used [15]. A confidence interval of 95% was considered for the proportion of intra-examiner concordance [15]. The Cronbach alpha was used for inter-examiner reliability of PFM strength scores, using TDP in the anterior and posterior areas [16]. The kappa test was used for inter- and intra-rater concordance of PFM strength, using TDP in the anterior and posterior areas [15]. Differences were considered statistically significant when p < 0.05.

## Results

The median ages were 35, 45, 54, and 67 years in the G1, G2, G3, and G4 age groups, respectively. There was a statistically significant difference between groups in age, BMI, number of pregnancies and vaginal delivery, as shown in Table 1. Of the 150 women, 69.3% reported sexual activity and in 40.7% reported regular physical activity, defined as occurring at least three times a week. There was a positive linear relationship between age and BMI (r = 0.188, p = 0.0212). There was a positive linear relationship between age and number of pregnancies (r = 0.265, p = 0.0010), and between age and vaginal deliveries (r = 0.258, p = 0.0014).

**Table 1:** **Demographic characteristic**

**Variables**	**GROUPS**				**Statistical analysis**
	**G1**	**G2**	**G3**	**G4**	
	**(n = 37)**	**(n = 39)**	**(n = 39)**	**(n = 35)**	
Age^(1)^	35.0 (30.0; 40.0)	45.0 (41.0; 50.0)	54.0 (50.0; 59.0)	67.0 (61.0; 86.0)^#^	p < 0.001
Body Mass Index^(2)^	24.9 (4.1)	26.5 (4.9)	25.7 (4.0)	28.0 (3.5)^##^	p = 0.015
Number of pregnancies^(1)^	3.0 (2.0; 7.0)	3.0 (2.0; 5.0)	3.0 (2.0; 8.0)	4.0 (2.0; 14.0)^##^	p = 0.015
Vaginal deliveries^(1)^	1.0 (0.0; 7.0)	1.0 (0.0; 3.0)	1.0 (0.0; 6.0)	2.0 (0.0; 8.0)^###^	p = 0.016

^(1)^Median (minimum value; maximum value).

^(2)^Mean (standard deviation).

^#^(p < 0.001) G4 xG3 xG2 xG1.

^##^(p < 0.05)G4 x G1.

^###^(p < 0.05) G4 x (G2,G3).

Considering the subjects graded as having mild contraction (Amaro grade 1), using TDP in both the anterior and posterior areas, there was a positive linear relationship between BMI and vaginal deliveries (r = 0.418, p = 0.013 and r = 0.302, p = 0.037, respectively). We observed no linear relationship between these factors in grades 2 and 3 of the PFM strength evaluation. There was no statistically significant difference in the different grades of PFM strength, in neither the anterior nor posterior areas, in relation to age (Table 2). There was good intra-rater concordance between anterior and posterior PFM assessments, being 64.7%, 63.3%, and 66.7% for examiners A, B, and C, respectively (Tables 3 and 4). The inter-rater concordance level was moderate to good, with kappa tests in the range of 0.523–0.736, between two examiners (Table 5).

**Table 2:** **Descriptive measures of ages according with grade of PFM strength using TDP assessment on anterior and posterior areas**

Grade Of Pfm strength*	**(n)**	**Anterior**	**(n)**	**Posterior**	**p value**
		**(age)** ^**(1)**^		**(age)** ^**(1)**^	
0	(5)	42 (33; 52)	(4)	38 (30; 43)	p = 0.190
1	(49)	48 (30; 73)	(36)	51 (30; 73)	p = 0.578
2	(71)	51 (30; 86)	(64)	48 (30; 86)	p = 0.611
3	(25)	49 (30; 72)	(46)	51 (30; 72)	p = 0.896
**p value**	(150)	p = 0.408	(150)	p = 0.123	

*Amaro’s classification [13].

^(1)^Median (minimum value; maximum value).

**Table 3:** **Intra- rater concordance between anterior and posterior transvaginal digital palpation (TDP) assessment and their respective confidence interval considering each examiner according Amaro’s Scale** [13 **]**

**Examiners**	**Concordance**	**Confidence interval**	
		**Lowerbound**	**Upperbound**
**A**	97/150 (64.7%)	57.0%	72.4%
**B**	95/150 (63.3%)	55.6%	71.0%
**C**	100/150 (66.7%)	59.2%	74.2%

**Table 4:** **Intra-rater concordance between anterior and posterior transvaginal digital palpation (TDP) assessment considering each examiner**

**Examiner**	**Transvaginal Digital Palpation (TDP)**
**A**	0.71*
**B**	0.67*
**C**	0.74*

*CONCORDANCE LEVEL (Kappa Test): 0–0.20: weak; 0.21-0.40: regular; 0.41-0.60: moderate; 0.61-0.80: good; >0.81: excellent.

**Table 5:** **Concordance level considering each two examiners in transvaginal digital palpation (TDP) assessment of PFM on anterior and posterior areas (Amaro’s classification)** [13 **]**

**Examiners**	**Transvaginal Digital Palpation (TDP)**	
	**Anterior**	**Posterior**
**AxB**	0.591*	0.571*
**AxC**	0.682*	0.736*
**BxC**	0.685*	0.523*

*CONCORDANCE LEVEL (Kappa Test): 0–0.20: weak; 0.21-0.40: regular; 0.41-0.60: moderate; 0.61-0.80: good; >0.81: excellent.

## Discussion

BMI was higher in the older age range, compared with younger women, and there was a progressive increase in BMI with aging. Other authors have also observed an increase in weight with aging and this factor could be correlated with menopause [17,18]. Different studies have demonstrated the presence of PFM dysfunction related to aging, parity, and vaginal deliveries [19,20]. Interestingly, in our series of continent women, despite the higher BMI and the higher number of pregnancies and vaginal deliveries in older women, there was no statistically significant difference in PFM strength in the different age ranges, showing that the aging process in continent women generally did not influence PFM strength. There was a positive linear relationship between PFM weakness, BMI, and vaginal deliveries though, and considering this, probably the interaction of these factors may have contributed to the decrease in PFM strength encountered in some of these continent women.

The International Continence Society (ICS) has defined by consensus, the diagnosis and treatment of pelvic floor dysfunctions [21]. They standardized the terminology of pelvic floor muscle function and acknowledged that assessing it by vaginal digital palpation is easy to perform, but emphasized that quantification of PFM contraction is problematic [21,22]. In our study, we used a scale of four grades, varying from 0 to 3, as described by Amaro et al. [13], with the objective to facilitate the understanding and reproducibility in clinical practice. However, different authors do not consider digital palpation of the vagina as a sensitive and reproducible method for the assessment of PFM function [11,23,24]. On the other hand, others have reported that this would be the best qualitative method to assess the contraction and muscular strength of PFM [11,25,26].

In our study, there was no correlation between muscle weakness and age. This finding is in agreement with the literature where the physiological aging "per se" in continent women does not correlate with decrease of PFM strength [27]. However, in incontinent women the PFM strength was significantly lower than continents and worsens during the aging process [3,28].

Our results are consistent with the literature that reports the difficulty of assessing PFM function by vaginal digital palpation, due to variability of its anatomy. This assessment still depends on the skill and experience of examiners. The examiners who participated in our study had 4–5 years of work experience after graduation and, despite that, there were some different interpretations of PFM contraction degree. Our find are in agreement with the literature, that shows reproducibility of the TDP method, with some restrictions [26,28-30]. Slieker-ten Hove et al. [31], conducted a reproducibility study with 4 different examiners by TDP, demonstrating high intra-observer rates of reproducibility, and low inter-examiner rates. According to the authors, the classifications used in the studies may not have enough accuracy to properly distinguish between individuals.

Morin et al. [30] reported that it is not possible to establish any correlation between TDP and objective methods of evaluation, such as dynamometer or perineometer. In another study of our group, we also observed that the correlation with objective methods of evaluation of PFM and its reproducibility are questionable [3,13].

The intra-rater reliability refers to the concordance of each anterior and posterior TDP assessment of pelvic floor contractions, for each subject and for each examiner. Our results objectively revealed a good level of concordance, indicating that the TDP assessment is accurate for evaluating the pelvic floor muscular strength in either position. However, when we take in consideration the inter-rater reliability between each two examiners, the concordance varied between moderate to good. Inter-rater reliability refers to the concordance of PFM grading on the same subject, by different examiners. This fact is in agreement with the findings of other authors that have highlighted the differential profile of vaginal pressure distributed along the vaginal canal [4], and that this is a subjective evaluation, dependent of examiners’ training [32]. Consequently, the accuracy of this assessment test depends on the skill and experience of the examining physical therapist.

Different measurement tools assess different aspects of PFM function, and it is important to look at them as complementary in a thorough PFM evaluation, not mutually exclusive. Further studies are necessary to evaluate the concordance between tests using different classifications and their inter-rater reliability.

## Conclusions

Age does not affect PFM strength profiles, in continent women. There is a good relationship between anterior and posterior vaginal PFM strength assessments, but only moderate to good inter-rater reliability of the measurements.

### Brief summary

This work intends to evaluate transvaginal palpation, as a clinical method to assess baseline strength of the pelvic floor, in multiparous continent women.

## Abbreviations

UI: Urinary incontinence
PFM: Pelvic floor muscle
ICIDH: International Classifications of Impairments, Disabilities and Handicaps
TDP: Transvaginal digital palpation
BMI: Body mass index
s: Seconds (s)
ICS: International Continence Society
